# Current News and Opinion

**Published:** 1887-04

**Authors:** 


### Current News and Opinion.

### CORRESPONDENCE.

*Editor Independent Practitioner:*

Will you kindly allow me a little space in your valuable journal. It is not often that I ask such a favor at your hands, for my extreme interest in and love for my fellow-man act as safeguards to protect your numerous readers from such inflictions. But there is one matter that I think should claim the attention of "the powers that be," and that, too, at the earliest possible moment. I would have preferred that it had been first suggested by some other than myself, but, as "everyone's business is nobody's business," it has been allowed to go this long unnoticed, at least in our dental journals.

As all are aware, the time is now rapidly approaching when the next meeting of the American Dental Association will convene—the first Tuesday in August next, in Asheville, North Carolina. It is also as well known that in the following month (September), the meeting of the International Medical Congress takes place at Washington, D C., in which a Section in Dental and Oral Surgery has been established; a Section, which those who have the matter in charge are bound shall in no way take a second place in the Congress. This invitation from the medical fraternity to take part with, and be one of their number at this time, may well be considered a high honor, no matter whether we may feel as some do, that dentistry and medicine are separate and distinct professions, or that our admission to this Congress is the last throe of parturition in our acknowledged birth into the medical ranks The fact is an established one, that we have had an invitation which has been *accepted.* The Section has been organized, and in the list of the officers appointed may be found the names of some of the best and brightest men in our profession.

But they alone cannot make the Section a success. Among the rank and file that it is hoped and expected will attend, should be found quite as many, or even more, of the same kind. That the American Dental Association will be largely represented here by its members will hardly admit of cavil or doubt, and now comes the point to which the above is but the prelude. *Will either the American Dental Association or the Dental Section of the Congress be apt to prove a complete success if both are held in the same year*, *and with so short a time intervening between the meetings?* I think that it can hardly be possible that such can be the case. There are few, very few, who could or would attend both these meetings, whilst many might attend one of them. Asheville is a long way off from the majority of those who attend the Association, and to go there would necessarily be an expensive trip in both time and money. Should any desire to attend both these meetings, they would scarcely have time to get home from one before they would be obliged to turn around and leave for the other.

My suggestion is that to make one success instead of two failures, it would be policy (and the word policy is hardly strong enough to express my meaning) to postpone the American Dental Association meeting for one year. Let the present officers of the Association hold over until 1888, when the meeting can be held at Asheville, or elsewhere, as the Executive Board may see fit. I am fully satisfied that the Association made a mistake in selecting that place for its next meeting, for I learned from good authority, while on a recent trip south, that there is no accommodation there for such numbers as usually attend the Association. There is a large and fine hotel (only one), kept by an old personal friend of mine, Mr C. N. Southwick. He says that he could make no reduction in rates at that time of the year, and that his house is full, and more than full at that season, so that we could not be accommodated with rooms.

Some have suggested that the meeting of the Association he held the week before the meeting of the Congress. This might be a trifle better than the way matters now stand, but not much. Two weeks in Washington, in August and September, would be a little too much for any others than those who are in training for a hotter climate than that of North Carolina, and I like to believe that I am not one of that number. The expense of two weeks at Washington at that time is also a matter that should be properly considered.

I have now spoken my little piece "right out in meetin'," and I trust that it may prove an entering wedge that shall start a movement that shall have for its end the "greatest good to the greatest number," which sentiment must be my apology for thus trespassing upon your pages.

Truly and fraternally yours,

DETROIT, March 21, 1887. GEO. L. FIELD.

---

*Editor Independent Practitioner:*

Will you allow me to request those of your readers who will assist in the Dental and Oral Section of the International Medical Congress to furnish me with an abstract of their papers or the papers themselves at once?

These papers must be in the hands of the secretaries as soon as possible, in order that a definite programme may be arranged and a proper amount of time apportioned for each subject. E. A. BOGUE, Secretary,

29 East 20th Street, New York City.

---

*Editor Independent Practitioner:*

The paper "Metal Plates," which you published in the March PRACTITIONER, has two annoying typographical blunders, which I wish you would please correct.

On page 134, 16th line, for *condition* read *contour*.

Then I am made to say in the same paragraph, "solder one end of the platinum loop," while it is necessary to solder both, unless it is soldered in the middle and the ends turned up, which is quite as well Simply spurring or etching the surface of the plate will sometimes hold, but is not reliable under much strain

L. P. HASKELL

### A NOVEL METHOD OF BLEACHING.

A case of office practice was recently related to me by Dr. A. H. Scofield, of New York, which is as follows :

In the mouth of a young lady was a discolored superior lateral incisor which she desired Dr. Scofield to treat with a view of restoring the color. Opening into the pulp-chamber through an old stopping on the posterior surface, the doctor found a filling in the root, apparently composed of fibres of cotton mixed with oxy-chloride of zinc. It was very compact, and over half an hour was spent in getting it out. After reaching almost to the end of the root, a small piece of the broach broke off and could not be dislodged. With the idea of getting rid of this bit of steel, the root canal was filled with crystals of iodine and sealed with gutta-percha. Next morning the lady appeared in a state of great alarm, for the whole tooth was as black as the iodine itself! The cavity was quickly opened and the iodine removed. Shreds of cotton wrapped around a broach and dipped in ninety per cent. alcohol were carried and re-carried into the pulp canal probably twenty or more times in as many minutes, then the root was packed with cotton soaked in alcohol, and the cavity again sealed. The following day, and again the day after, the washing process was repeated, each time the tooth looking better, and after this last treatment the lady was requested to allow it to so remain three days. She called at the time appointed, and the tooth was entirely free from discoloration—as clear and white as before it became devitalized. C. E. Francis.

### EIGHTH DISTRICT DENTAL SOCIETY.

The nineteenth annual meeting of the above organization will occur in Buffalo, on Tuesday and Wednesday, April 19th and 20th. The Society will convene at The Genesee at half past ten o'clock A. M. of the first day, and the sessions will be continued throughout that and the next day until the routine of business and the programme as arranged by the business committee are completed.

The business of the Society requiring attention is of more or less interest and importance to the members, and the topics which have been selected for discussion certainly should elicit much valuable thought and profitable comment. The list includes the following subjects :

Dentistry versus Medicine. A review; by H. A. Birdsall, D. D. S.

Neuralgia; by W. C. Hayes, M. D. S.

Restoration of Discolored Teeth; by W. A Barrows, D. D. S.

Soft Gold in Filling Teeth; by B. Rathbun, M. D. S.

Thermometer Tests in Vulcanizing, with practical demonstrations; by G. B. Snow, D. D. S.

Failures; by M. H. Dailey.

The Dividing Line; by F. W. Low.

In addition to this programme, the relating of Incidents of Office Practice, and, if time will permit, Demonstrations in Operative Dentistry, will occupy the attention of the convention.

Every dental practitioner in the district will be welcome and cordially received. It is the aim of the Business Committee to make the meeting as profitable, interesting and attractive as the circumstances will permit.

### NEW YORK COLLEGE OF DENTISTRY.

The twenty-first annual commencement of the New York College of Dentistry was held at Chickering Hall, Wednesday evening, March 9, 1887.

### GRADUATES.

James Charles Alker.
George Sumner Burt.
Gregorio Santos Benet y Llata, M. D.
Samuel Skinner Brown.
Valentine Edw. Norman Cook.
Thomas Alfred Clawson.
John Harvey Crane.
John Richard Crawford.
John Clayton Downs.
Frank Perry Denny.
George Anthony Dow.
Frank John Eversfield.
William Eybel,
Samuel Hassell, Jr.
Erastus Otis Houghton.
Spencer Cone Hamilton.
Paul William Hiller
Halstead Pell Hodson.
Ira Daniel Horton.
Samuel Porter Hopkins.
Leo Frederic Hugle.
Frank Alfred Katzmeier.
Samuel James Kennedy.
Louis Charles Leroy.
Frank Butler Longnecker.
Edwin Parker Marshall.
Francis Joseph McLaren.
Ferdinand Moith.
Henry Middleton.
Lorenzo Noa.
George Edward Nearing.
Arthur German Rouse.
Franklin Willard Rogers.
Dudley John Russell.
Horace Reynolds.
Felix Edmond San Fuentes, Ph. B.
Thomas Howard Stevens.
Charles Harvey Smith.
Harold Slade.
Preston McCready Sharp.
Richard James Secor.
Walter Lincoln Scofield.
Joseph Daniel Sayre.
George Joseph Taylor.
Daniel Webster Valentine.
Walter Woolsey.
Herman Eugen Albert Wichert.
George Mortimer Whitfield.
John Van Pelt Wicks.
Ulysses Grant Woolley.
Leonhardt Eichbery Zuchtmann.

Number of matriculates 193.

---

### ANOTHER NEW DENTAL COLLEGE.

A dental department has been organized in the new Medical College of Buffalo—Niagara University. The faculty is not complete at present, but the following have been appointed:

Dr. A. P. Southwick, professor of Operative Dentistry and Oral Deformities.

Dr. S. Eschelman, professor of Histology and Microscopy.

Dr. George B. Snow, professor of Mechanical Dentistry and Metallurgy.

Dr. F. E. Howard, professor of Operative Dentistry, Hygiene, and Constructive Dentistry.

Dr. C. A. Allen, professor of Materia Medica, Therapeutics and Prosthetic Dentistry.

Dr. Theodore G. Lewis, Secretary and Demonstrator of Dental Art and Mechanism.

### OHIO COLLEGE OF DENTAL SURGERY.

The forty-first annual commencement of the Ohio College of Dental Surgery was held in College Hall, Cincinnati, Ohio, Wednesday evening, March 2, 1887.

GRADUATES.

A. W. Black...............Indiana.
L. A. Brown............ Minnesota.
L. E. Custer..................Ohio.
L. E. Day.......................Ohio.
G. H. Doulton............California.
W. F. Edmonds..........Kentucky.
J. S. Emery....................Ohio.
J. W. Fisher.............Kentucky.
C. H. Green, Jr..............Indiana.
E. S. Griffis....................Ohio.
M. A. Hadcock.............Canada.
B. C. Hinkley..................Ohio.
T. L. Johnson.................Ohio.
E. S. Keefer...................Ohio.
Miss M. L. Leininger...........Ohio.
C. H. Martin......................Ohio.
E. S. Mathews.............England.
H. C. Matlack............Kentucky.
E. J. McCartney......Pennsylvania.
B. A. McGee ..............Indiana.
C. E. Miles.......................Ohio.
A. H. Millman...................Ohio.
B. A. Mosbey...............Indiana.
W. W. Reed.....................Ohio.
F. L Rice..........................Ohio.
J. M. Rutherford...............Ohio.
E. J. Schwartz..................Ohio.
James Silcott....................Ohio.
J. J. Werner...........Switzerland.

### FIFTH DISTRICT DENTAL SOCIETY.

The Fifth District Dental Society of the State of New York will hold its nineteenth annual meeting, at Utica, Tuesday and Wednesday, April 12th and 13th, 1887.

The session will be called to order at 2 P. M. at the St. James, Whitesboro Street, near Genesee.

Applications for membership in the Society should be made on or before the day of meeting, to the Chairman of the Board of Censors, or the Recording Secretary.

The Board of Censors will be in attendance to examine candidates for admission to the Society.

Members of the profession from other societies are cordially invited to be present and take part in the discussions.

C. J. PETERS, Rec. Sec

### MINNESOTA HOSPITAL COLLEGE.

The fifth annual commencement of the Dental Department of the Minnesota Hospital College was held in connection with the medical department, in the Hennepin Av. M. E. Church, Minneapolis, Minn., on Friday evening, March 11th, 1887. The address was delivered to the graduates by Prof. John E. Bradley, and the valedictory by James E. Cummings, D. D. S.

The number of matriculates for the session was nineteen.

The degree of D. D. S. was conferred on the following graduates by C. H. Hunter, A. M , M. D., President of the Faculty :

Thomas S. Rounce........Wisconsin.

James E. Cummings......New York.

Clarence Strachauer......Minnesota.

Horace B. Ober.........Connecticut.

### CONNECTICUT VALLEY DENTAL SOCIETY.

The semi-annual meeting of this society will be held in Montreal, P. Q., July 19th to 22d, inclusive

Prof. G. V. Black, M. D., D. D. S., of Jacksonville, and Prof. Chas. Mayr, of Springfield, Mass., will be the guests of the society, and will present subjects of interest to the profession. Interesting papers have also been promised by Prof. R. R. Andrews, of Cambridge, Dr. James McManus, of Hartford, Dr. Geo. A. Maxfield, of Holyoke, Dr. F. F. Trestler, Dr. Geo. W. Beers and Dr. Geo. H. Weagert, of Montreal.

There will be but one session of the society each day, and that in the morning. The afternoons and evenings will be devoted to excursions, social pleasures, etc., under the charge of a committee of Montreal gentlemen. To say that Dr. Lovejoy is chairman of this committee, is a sufficient guarantee that nothing will be left undone that will add to the comfort and pleasure of all.

GEO. A. MAXFIELD, D. D S., Sec.

---

### SEVENTH DISTRICT DENTAL SOCIETY.

The Seventh District Dental Society of the State of New York will hold its nineteenth annual convention in Rochester, on Tuesday and Wednesday, April 26th and 27th.

Dentists of the Seventh Judicial District of the State who wish to become members of the society should make application to the Chairman of the Board of Censors, Dr. H. C. Knickerbocker, Seneca Falls, before noon of the first day of the convention.

An interesting programme is being arranged.

Members of the profession are invited to be present.

CHAS. T. HOWARD, Rec. Sec.

224 E. Main St., Rochester, N. Y.

---

### ILLINOIS STATE DENTAL SOCIETY.

The twenty-third annual meeting of the Illinois State Dental Society will be held at Jacksonville, beginning Tuesday, May 10th, and continuing four days.

A full programme of scientific work is arranged for, including a large and instructive clinic.

Dr. Black will give short lectures on micro-organisms, with practical demonstrations of their culture. All dentists will be cordially welcomed.

J. W. WASSALL, Secretary.

---

DR. W. H. WAITE, of Liverpool, who is well known in America, has been obliged, through failure of sight, to relinquish the active practice of dentistry. His English friends and admirers have determined to present him with a testimonial, in the shape of a purse of gold and an address on vellum. The success of the attempt is assured by the consent of such men as Sir Edwin Saunders, Dentist to the Queen, Sir John Tomes, J. S. Turner, C S. Tomes, Thos. Underwood, Felix Weiss, and others, the best known of English dentists, to serve upon the committee.

DRUMINE is the name of a new Australian local anæsthetic discovered and described by Dr. John Reid, of Port Germain, South Australia. *Euphorbia Drummondii* is the species, from the milky juice of which the alkaloid Drumine was prepared. Cocaine is known to have a mixed action on sensory and motor nerves, and causes preliminary excitement; while drumine is said to have an almost purely sensory paralyzing effect, and does not cause excitement. Experiments were made on cats and on the observer's tongue. It was injected into the legs of the former animals and caused much dullness, with marked impairment, apparently, of all forms of sensibility. Placed on the tongue, nostrils and hand of the observer, the resulting anæsthesia was most marked. The alkaloid has no action on the pupil, and small doses given internally produce no constitutional effect. It has been employed successfully in subcutaneous injections for sciatica and sprains. The experimentation has, so far, been imperfect and incomplete. We hope soon to have a fuller account of this new alkaloid, and to be able to give further information thereon. The amount injected subcutaneously was four minims of a four per cent. solution. Dr. Reid anticipates a brilliant future for the drug in the domain of nervous and cerebral diseases.—*London Lancet.*

---

PATIENT :—"Doctor, I have a very troublesome tooth. I must have something done for it at once."

Young Dentist—"Um ! Well, let me see. I cannot give you any time today. Every moment is taken, but—John, hand me my appointment book. Oh, yes ; I can give you the day after to-morrow at ten o'clock."

Patient—"But I can't come then, and my tooth troubles me constantly."

Y. D.—"I am very sorry, but that is my first open date. If you cannot come then, I can make it the day after at twelve, by giving you my lunch hour."

Patient—"Well, I suppose that must do then."

Y. D.—(To his office boy after the patient's departure). "John, here is my last quarter. Run out and get some bread and cheese, and we will try and worry through on it. It was necessary that I give her the impression of a rush of business or she would never come back again."

---

A MIXTURE of one part of iodoform to ten or fifteen of collodion, if spread upon a neuralgic surface until it attains a thickness of one or two millimetres, is said to be quite effective in the treatment of certain neuralgias. If the first application does not speedily terminate the neuralgia, those who have used this mode of treatment direct that its application should be continued. It seems especially valuable in the relief of neuralgias of the trigeminus. It also seems of value to be applied along the spine, particularly at painful points in what is called spinal irritation. These observations are by no means new, and yet they seem worthy of further consideration.—*Neurological Review.*

---

R. S. WILLIAMS' advertisement, upon the fourth cover page, was received last month at the last moment, and in setting it up several errors were made. They are corrected in this number. By the way, see what an array of different preparations of gold Mr. Williams presents.

Reduced rates have been secured on certain lines of ocean steamers for members and their families who will attend the Medical Congress this summer, as follows :

Inman Line, Liverpool to New York and return, $100,00.
Red Star Line, Antwerp to New York and return, $100.00.
Hamburgh Line, Hamburgh to New York and return, $90.00.
Royal Netherland, Amsterdam to New York and return, $80.00.
The Cunard and White Star Lines decline to make any reductions.

---

In the filling of pulp canals with liquid gutta-percha, it is necessary that they be kept completely free from saliva and moisture that the material may penetrate to the remotest end. It cannot be introduced into a wet canal. To keep the instrument clean and to prevent the solution from sticking, it may be occasionally dipped in alcohol. If the canal be wiped out with alcohol, the gutta-percha solution will flow better. The gutta-percha points should not be used until the canal is first filled with the gutta-percha solution, as they will not usually penetrate to the foramen.

---

Dr. C. E. Klotz, of St Catharines, Canada, has devised an excellent implement for cutting sand or emery paper into strips for polishing teeth and fillings. It consists of a circular disk, an inch and a half in diameter, beveled upon one side to a sharp edge, and revolving in one end of a split shaft of iron, like a tracing wheel. It runs beside a straight-edge placed upon the sheet of sand or emery paper, this resting, paper side up, upon a piece of soft wood. It cuts the strips cleanly and evenly, and is self-sharpening.

---

It is confidently predicted that in five years the magnesium light will be as familiar as is the electric light to day. Its high cost has heretofore been a serious obstacle, but that is said to be now removed by a new German process, which has reduced the price from $40 to $8 a pound, with a prospect of still further cheapening it. A wire of moderate size equals the light of seventy-five stearine candles; the cost is now but little more than gas.—*National Druggist.*

---

On the planet Jupiter, according to Swedenborg, men live to an age equal to about thirty of our years They become bald at the age of twenty-nine, and knowing this to be the sure precursor of their death within a year, they instantly set about preparing themselves for that event. When we remember how men act on this miserable old earth after they become bald, we don't see how they can ever look at Jupiter without blushing for shame.—*Leavenworth Times.*

---

The American coins below the denomination of the twenty-five cent piece are the twenty cent piece, the dime, the half-dime, the three cent piece, the two cent piece, and the cent. Using only these, the twenty-five cent piece may be changed into other coinage in 215 different ways.

---

The Medical Register is a new medical weekly journal, published in Philadelphia, at $3.00 per annum It is edited by Drs. John V. Shoemaker and William C. Wile, both of whom have made their mark in medical literature.

ARCHIVES OF DENTISTRY celebrates the beginning of its fourth volume by a change in the editorial management. Dr. W. H. Eames, formerly editor of *The Missouri Dental Journal*, and Dr. C. T. Stockwell, formerly editor of the *New England Dental Journal*, assume the management, with Dr. John G. Harper as associate. Under their vigorous direction we shall expect to see *Archives* become even better than it was before.

P. S. Forty-seven more editors and thirty correspondents are appointed.

---

A DIPLOMA MILL was discovered in Lewiston, Me., under the name of the "Druidic University of America, State of Maine Branch." The "University" is an incorporated institution, with a faculty composed of one member, who styles himself "Dr. Samuel York, Druidic Physician." Since the exposure the Maine Legislature has repealed the charter of the Druidic University.

---

DR. F. P. VANDENBURG, of Buffalo, has been appointed city chemist. The ordinances provide that this officer shall hold the degree of B. S., or its equivalent. The city attorney declared Dr. Vandenburg's M. D. an equivalent, while the opponents of the appointee claim that only Ph. B. and Ph. D. are equivalent to B. S. But Dr. Vandenburg is city chemist all the same.

---

THE BILL making an appropriation for the International Medical Congress, which, when it first came before the United States Congress, named $50,000 as the sum to be given, has been passed, with some amendments, one of which cuts the amount down to $10,000.

---

PARKE, DAVIS & CO.'s advertisement was crowded out of our last issue, but again appears on an inside page. We hope our readers will notice their list of specialties which are specially prepared for dentist's use.

---

A PHYSICIANS' AND DENTISTS' INSURANCE ASSOCIATION has been incorporated in Illinois. In its executive committee and advisory board may be found the names of some of the best-known dentists of the west.

---

TECHNICS is a beautifully printed and well edited bi-weekly medical journal, published in Boston, and edited by Drs. Jos. H. and Chas. Everett Warren. Its contents are of an exceedingly practical character.

---

OLIVER WENDELL HOLMES, during his tour abroad, had conferred upon him honorary degrees as follows: D. C. L., Oxford; Litt. Doc., Cambridge; LL. D., Edinburgh; LL. D., Dublin.

---

DR. T. B. WHEELER, of Chicago, proposes to locate in Paris about May 1. He has sold out his home practice, and will turn his face towards the rising instead of the setting sun.

---

THE DENTAL RECORD says that Sir John Tomes, F. R. S., is very ill with pleuro-pneumonia, which at his advanced age makes recovery a matter of doubt.

---

FIRM PRESSURE made with the thumbs on the supra-orbital nerves will cut short an attack of hysteria

---

BILLROTH, the well known surgeon and pathologist, of Vienna, has been elevated to the peerage.

